# 2-Chloro-*N*-(2-methyl­phen­yl)benzamide

**DOI:** 10.1107/S1600536812023902

**Published:** 2012-05-31

**Authors:** Vinola Z. Rodrigues, B. Thimme Gowda, Július Sivý, Viktor Vrábel, Jozef Kožíšek

**Affiliations:** a Department of Chemistry, Mangalore University, Mangalagangotri-574 199, Mangalore, India; b Institute of Mathematics and Physics, Faculty of Mechanical Engineering, Slovak University of Technology, Námestie slobody 17, SK-812 37 Bratislava, Slovak Republic; c Institute of Analytical Chemistry, Faculty of Chemical and Food Technology, Slovak University of Technology, Radlinskeho 9, SK-812 37 Bratislava, Slovak Republic; d Institute of Physical Chemistry and Chemical Physics, Faculty of Chemical and Food Technology, Slovak University of Technology, Radlinského 9, SK-812 37 Bratislava, Slovak Republic

## Abstract

In the title compound, C_14_H_12_ClNO, the two aromatic rings are almost coplanar, making a dihedral angle of 4.08 (18)°. In the crystal, N—H⋯O hydrogen bonds link the mol­ecules into infinite chains running along the *a* axis.

## Related literature

For studies on the effects of substituents on the structures and other aspects of *N*-(ar­yl)-amides, see: Bowes *et al.* (2003 ▶); Gowda *et al.* (2000 ▶); Rodrigues *et al.* (2012 ▶); Saeed *et al.* (2010 ▶) of *N*-chloro­aryl­amides, see: Gowda & Rao (1989 ▶); Jyothi & Gowda (2004 ▶) and of *N*-bromo­aryl­sulfonamides, see: Gowda & Mahadevappa (1983 ▶); Usha & Gowda (2006 ▶).
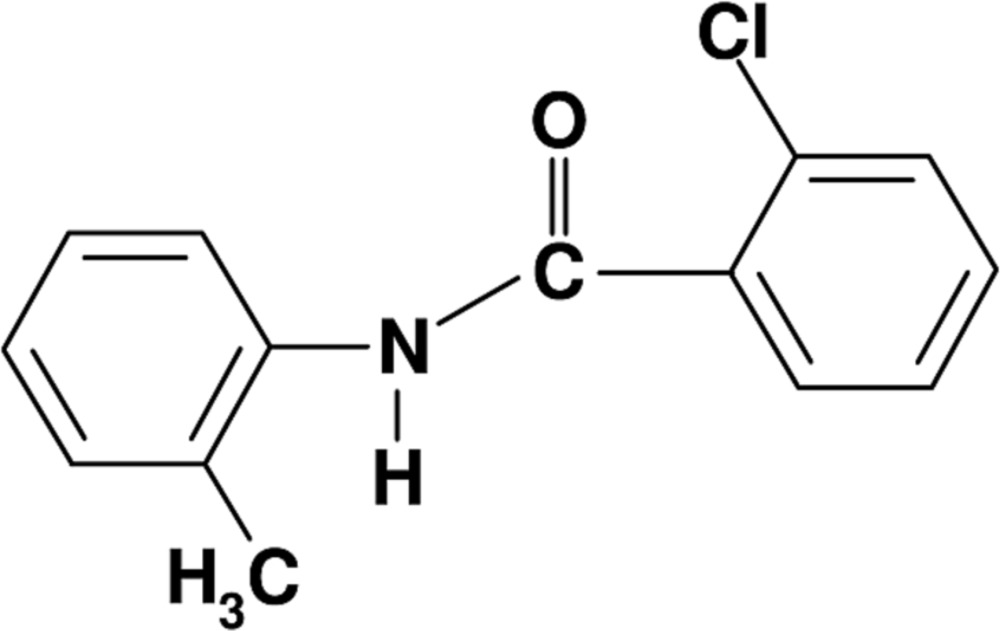


## Experimental

### 

#### Crystal data

C_14_H_12_ClNO
*M* *_r_* = 245.70
Orthorhombic, 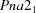

*a* = 9.746 (3) Å
*b* = 6.077 (3) Å
*c* = 20.797 (7) Å
*V* = 1231.8 (8) Å^3^
*Z* = 4
Mo *K*α radiation
μ = 0.29 mm^−1^
*T* = 295 K
0.55 × 0.40 × 0.25 mm

#### Data collection

Oxford Diffraction Xcalibur Ruby Gemini diffractometer
Absorption correction: analytical [*CrysAlis RED* (Oxford Diffraction, 2009 ▶), based on expressions derived by Clark & Reid (1995 ▶)] *T*_min_ = 0.865, *T*_max_ = 0.921
16249 measured reflections
2173 independent reflections
1369 reflections with *I* > 2σ(*I*)
*R*_int_ = 0.097

#### Refinement

*R*[*F* ^2^ > 2σ(*F* ^2^)] = 0.068
*wR*(*F* ^2^) = 0.147
*S* = 1.10
2173 reflections
158 parameters
2 restraints
H atoms treated by a mixture of independent and constrained refinement
Δρ_max_ = 0.22 e Å^−3^
Δρ_min_ = −0.14 e Å^−3^
Absolute structure: Flack (1983 ▶), 1054 Friedel pairs
Flack parameter: 0.37 (13)

### 

Data collection: *CrysAlis CCD* (Oxford Diffraction, 2009 ▶); cell refinement: *CrysAlis CCD*; data reduction: *CrysAlis RED* (Oxford Diffraction, 2009 ▶); program(s) used to solve structure: *SHELXS97* (Sheldrick, 2008 ▶); program(s) used to refine structure: *SHELXL97* (Sheldrick, 2008 ▶); molecular graphics: *DIAMOND* (Brandenburg, 2002 ▶); software used to prepare material for publication: *SHELXL97*, *PLATON* (Spek, 2009 ▶) and *WinGX* (Farrugia, 1999 ▶).

## Supplementary Material

Crystal structure: contains datablock(s) I, global. DOI: 10.1107/S1600536812023902/bt5935sup1.cif

Structure factors: contains datablock(s) I. DOI: 10.1107/S1600536812023902/bt5935Isup2.hkl

Supplementary material file. DOI: 10.1107/S1600536812023902/bt5935Isup3.cml

Additional supplementary materials:  crystallographic information; 3D view; checkCIF report

## Figures and Tables

**Table 1:** Hydrogen-bond geometry (Å, °)

*D*—H⋯*A*	*D*—H	H⋯*A*	*D*⋯*A*	*D*—H⋯*A*
N1—H1⋯O1^i^	0.86 (1)	2.00 (1)	2.853 (5)	171 (5)

Symmetry code: (i) 
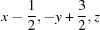
.

**Fig. 1.**
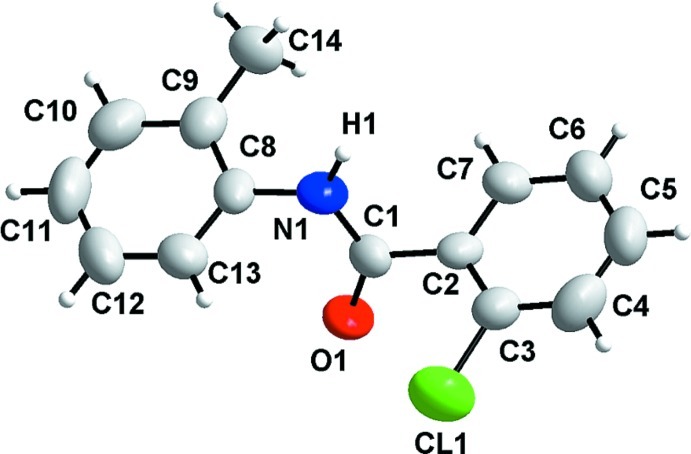
Molecular structure of the title compound with the atomic numbering scheme. Displacement ellipsoids are drawn at the 50% probability level. H atoms are represented as small spheres of arbitrary radii.

**Fig. 2.**
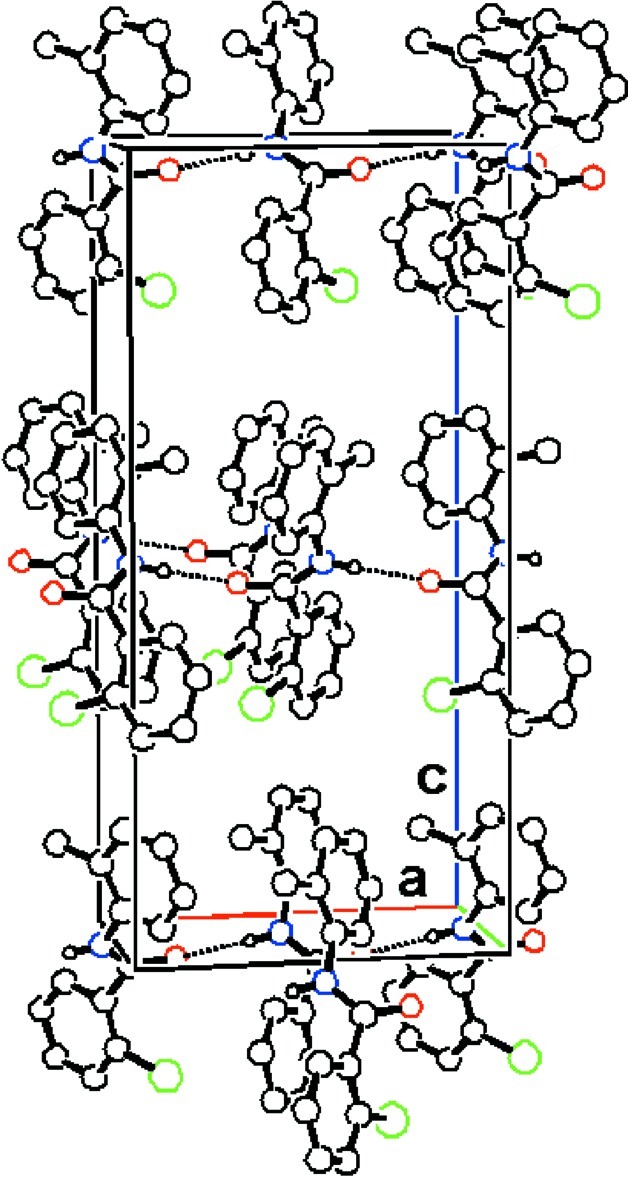
Packing view of the title compound. Molecular links along a-axis are generated by N–H···O hydrogen bonds which are shown by dashed lines. H atoms have been omitted for clarity.

## supplementary crystallographic information

### Comment

The amide and sulfonamide moieties are the constituents of many biologically important compounds. As part of our studies on the substituent effects on the structures and other aspects of *N*-(aryl)-amides (Bowes *et al.*, 2003; Gowda *et al.*, 2000; Rodrigues *et al.*, 2012; Saeed *et al.*, 2010), *N*-chloroarylsulfonamides(Gowda & Rao, 1989; Jyothi & Gowda, 2004) and *N*-bromoarylsulfonamides(Gowda & Mahadevappa, 1983; Usha & Gowda, 2006), in the present work, the crystal structure of 2-chloro-*N*-(2-methylphenyl)benzamide has been determined (Fig.1).

In the title compound, the *ortho*-Cl atom in the benzoyl ring is positioned *syn* to the C=O bond, similar to that observed in 2-chloro-*N*-(3-methylphenyl)benzamide (I) (Rodrigues *et al.*, 2012). The *ortho*-methyl group in the anilino ring is also positioned *syn* to the N—H bond, in contrast to the *anti* conformation observed between the *meta*-methyl group and the N—H bond in (I).

The central amide core –NH—C(=O)– group is twisted by 58.77 (27)° and 56.30 (28)° out of the planes of the 2-chlorophenyl and 2-methylphenyl rings, respectively, while the two aromatic rings make only a dihedral angle of 4.08 (18)°, compared to the value of 38.7 (1)° in (I)

In the crystal structure, intermolecular N—H···O hydrogen bonds (Table 1) link the molecules into infinite chains running along the *a*-axis. Part of the crystal structure is shown in Fig. 2.

### Experimental

The title compound was prepared by the method similar to the one described by Gowda *et al.* (2000). The purity of the compound was checked by determining its melting point. It was characterized by recording its infrared and NMR spectra.

Plate like colorless single crystals of the title compound used in X-ray diffraction studies were obtained by slow evaporation of an ethanol solution of the compound (0.5 g in about 30 ml of ethanol) at room temperature.

### Refinement

Hydrogen atoms were placed in calculated positions with C–H distances of 0.93 Å (C-aromatic), 0.96 Å (*C*-methyl) and constrained to ride on their parent atoms. The amide H atom was visible in a difference map and refined with the N—H distance restrained to 0.860 (2) Å. The *U*iso(H) values were set at 1.2*U*eq (C-aromatic) or 1.5*U*eq (C-methyl).

### Crystal data

**Table:**

C_14_H_12_ClNO	*F*(000) = 512
*M**_r_* = 245.70	*D*_x_ = 1.325 Mg m^−^^3^
Orthorhombic, *P**n**a*2_1_	Mo *K*α radiation, λ = 0.71073 Å
Hall symbol: P 2c -2n	Cell parameters from 2182 reflections
*a* = 9.746 (3) Å	θ = 3.5–25.0°
*b* = 6.077 (3) Å	µ = 0.29 mm^−^^1^
*c* = 20.797 (7) Å	*T* = 295 K
*V* = 1231.8 (8) Å^3^	Plate, colourless
*Z* = 4	0.55 × 0.40 × 0.25 mm

### Data collection

**Table:**

Xcalibur, Ruby, Gemini diffractometer	2173 independent reflections
Radiation source: fine-focus sealed tube	1369 reflections with *I* > 2σ(*I*)
Graphite monochromator	*R*_int_ = 0.097
Detector resolution: 10.434 pixels mm^-1^	θ_max_ = 25.0°, θ_min_ = 3.5°
ω scans	*h* = −11→11
Absorption correction: analytical [*CrysAlis RED* (Oxford Diffraction, 2009), based on expressions derived by Clark & Reid (1995)]	*k* = −7→7
*T*_min_ = 0.865, *T*_max_ = 0.921	*l* = −24→24
16249 measured reflections	

### Refinement

**Table:**

Refinement on *F*^2^	Secondary atom site location: difference Fourier map
Least-squares matrix: full	Hydrogen site location: inferred from neighbouring sites
*R*[*F*^2^ > 2σ(*F*^2^)] = 0.068	H atoms treated by a mixture of independent and constrained refinement
*wR*(*F*^2^) = 0.147	*w* = 1/[σ^2^(*F*_o_^2^) + (0.058*P*)^2^] where *P* = (*F*_o_^2^ + 2*F*_c_^2^)/3
*S* = 1.10	(Δ/σ)_max_ < 0.001
2173 reflections	Δρ_max_ = 0.22 e Å^−^^3^
158 parameters	Δρ_min_ = −0.14 e Å^−^^3^
2 restraints	Absolute structure: Flack (1983), 1054 Friedel pairs
Primary atom site location: structure-invariant direct methods	Flack parameter: 0.37 (13)

### Special details

**Table:**

Geometry. All e.s.d.'s (except the e.s.d. in the dihedral angle between two l.s. planes) are estimated using the full covariance matrix. The cell e.s.d.'s are taken into account individually in the estimation of e.s.d.'s in distances, angles and torsion angles; correlations between e.s.d.'s in cell parameters are only used when they are defined by crystal symmetry. An approximate (isotropic) treatment of cell e.s.d.'s is used for estimating e.s.d.'s involving l.s. planes.
Refinement. Refinement of *F*^2^ against ALL reflections. The weighted *R*-factor *wR* and goodness of fit *S* are based on *F*^2^, conventional *R*-factors *R* are based on *F*, with *F* set to zero for negative *F*^2^. The threshold expression of *F*^2^ > σ(*F*^2^) is used only for calculating *R*-factors(gt) *etc*. and is not relevant to the choice of reflections for refinement. *R*-factors based on *F*^2^ are statistically about twice as large as those based on *F*, and *R*- factors based on ALL data will be even larger.

### Fractional atomic coordinates and isotropic or equivalent isotropic displacement parameters (Å^2^)

**Table:**

	*x*	*y*	*z*	*U*_iso_*/*U*_eq_	
C1	0.5638 (4)	0.6837 (8)	0.4527 (3)	0.0506 (12)	
C2	0.5037 (4)	0.5445 (10)	0.3997 (2)	0.0497 (14)	
C3	0.5364 (4)	0.5696 (9)	0.3373 (3)	0.0596 (15)	
C4	0.4845 (7)	0.4319 (12)	0.2916 (3)	0.0833 (19)	
H4A	0.5099	0.4492	0.2488	0.100*	
C5	0.3941 (6)	0.2667 (12)	0.3087 (5)	0.0850 (17)	
H5A	0.3587	0.1723	0.2777	0.102*	
C6	0.3578 (5)	0.2439 (10)	0.3713 (4)	0.0784 (17)	
H6A	0.2968	0.1338	0.3834	0.094*	
C7	0.4101 (4)	0.3811 (10)	0.4159 (3)	0.0644 (16)	
H7A	0.3830	0.3660	0.4585	0.077*	
C8	0.5196 (4)	0.9375 (9)	0.5395 (3)	0.0552 (14)	
C9	0.4657 (5)	0.9069 (10)	0.6004 (3)	0.0646 (16)	
C10	0.5118 (6)	1.0562 (13)	0.6480 (3)	0.0815 (18)	
H10A	0.4756	1.0470	0.6893	0.098*	
C11	0.6062 (7)	1.2098 (13)	0.6351 (4)	0.096 (3)	
H11A	0.6403	1.2963	0.6684	0.115*	
C12	0.6535 (5)	1.2420 (9)	0.5735 (4)	0.0813 (19)	
H12A	0.7152	1.3549	0.5647	0.098*	
C13	0.6101 (4)	1.1096 (10)	0.5260 (3)	0.0679 (17)	
H13A	0.6402	1.1324	0.4841	0.082*	
C14	0.3642 (5)	0.7289 (9)	0.6169 (3)	0.0664 (16)	
H14C	0.3400	0.7395	0.6615	0.100*	
H14B	0.4044	0.5875	0.6086	0.100*	
H14A	0.2833	0.7461	0.5910	0.100*	
N1	0.4759 (4)	0.7929 (8)	0.4882 (2)	0.0611 (12)	
H1	0.3902 (14)	0.785 (9)	0.479 (3)	0.070 (17)*	
O1	0.6897 (3)	0.6844 (6)	0.46020 (18)	0.0739 (11)	
Cl1	0.64973 (16)	0.7710 (3)	0.31282 (10)	0.0971 (6)	

### Atomic displacement parameters (Å^2^)

**Table:**

	*U*^11^	*U*^22^	*U*^33^	*U*^12^	*U*^13^	*U*^23^
C1	0.042 (2)	0.046 (3)	0.063 (3)	0.001 (2)	−0.005 (2)	−0.001 (3)
C2	0.029 (2)	0.071 (4)	0.049 (3)	0.006 (2)	−0.002 (2)	−0.002 (2)
C3	0.044 (3)	0.076 (4)	0.059 (4)	0.006 (2)	0.000 (3)	−0.003 (3)
C4	0.088 (4)	0.102 (6)	0.060 (4)	0.024 (4)	−0.003 (3)	−0.011 (4)
C5	0.076 (4)	0.082 (4)	0.097 (5)	0.004 (3)	−0.012 (4)	−0.035 (4)
C6	0.064 (4)	0.078 (5)	0.093 (5)	−0.010 (3)	−0.004 (3)	−0.020 (4)
C7	0.042 (3)	0.081 (4)	0.070 (4)	−0.004 (2)	0.001 (2)	−0.004 (3)
C8	0.036 (2)	0.063 (4)	0.067 (4)	0.010 (3)	−0.011 (3)	−0.017 (3)
C9	0.054 (3)	0.074 (4)	0.066 (4)	0.020 (3)	−0.015 (3)	−0.011 (4)
C10	0.065 (3)	0.114 (6)	0.065 (4)	0.020 (4)	−0.002 (3)	−0.010 (4)
C11	0.090 (5)	0.104 (6)	0.094 (6)	0.015 (4)	−0.028 (4)	−0.049 (5)
C12	0.061 (3)	0.067 (4)	0.116 (6)	0.001 (3)	−0.015 (3)	−0.023 (4)
C13	0.046 (3)	0.077 (5)	0.080 (4)	0.002 (3)	−0.003 (3)	−0.006 (4)
C14	0.059 (3)	0.069 (4)	0.071 (4)	−0.001 (3)	−0.006 (2)	0.015 (3)
N1	0.039 (2)	0.082 (3)	0.062 (3)	0.002 (2)	−0.005 (2)	0.002 (3)
O1	0.0306 (15)	0.100 (3)	0.091 (2)	0.0024 (15)	−0.0065 (16)	−0.020 (2)
Cl1	0.0948 (11)	0.1063 (14)	0.0900 (10)	−0.0151 (10)	0.0021 (11)	0.0281 (10)

### Geometric parameters (Å, º)

**Table:**

C1—O1	1.237 (5)	C8—C13	1.396 (7)
C1—N1	1.311 (6)	C8—N1	1.446 (6)
C1—C2	1.507 (7)	C9—C10	1.416 (8)
C2—C3	1.346 (6)	C9—C14	1.506 (8)
C2—C7	1.389 (7)	C10—C11	1.337 (10)
C3—C4	1.364 (7)	C10—H10A	0.9300
C3—Cl1	1.725 (6)	C11—C12	1.377 (10)
C4—C5	1.382 (9)	C11—H11A	0.9300
C4—H4A	0.9300	C12—C13	1.342 (8)
C5—C6	1.356 (11)	C12—H12A	0.9300
C5—H5A	0.9300	C13—H13A	0.9300
C6—C7	1.347 (8)	C14—H14C	0.9600
C6—H6A	0.9300	C14—H14B	0.9600
C7—H7A	0.9300	C14—H14A	0.9600
C8—C9	1.384 (7)	N1—H1	0.861 (2)
			
O1—C1—N1	125.1 (4)	C8—C9—C10	115.7 (6)
O1—C1—C2	118.7 (4)	C8—C9—C14	123.7 (5)
N1—C1—C2	116.2 (4)	C10—C9—C14	120.6 (5)
C3—C2—C7	118.0 (5)	C11—C10—C9	121.7 (6)
C3—C2—C1	123.3 (5)	C11—C10—H10A	119.2
C7—C2—C1	118.7 (5)	C9—C10—H10A	119.2
C2—C3—C4	121.0 (5)	C10—C11—C12	121.1 (7)
C2—C3—Cl1	121.1 (4)	C10—C11—H11A	119.5
C4—C3—Cl1	117.9 (5)	C12—C11—H11A	119.5
C3—C4—C5	120.2 (6)	C13—C12—C11	119.6 (6)
C3—C4—H4A	119.9	C13—C12—H12A	120.2
C5—C4—H4A	119.9	C11—C12—H12A	120.2
C6—C5—C4	119.2 (7)	C12—C13—C8	120.1 (6)
C6—C5—H5A	120.4	C12—C13—H13A	120.0
C4—C5—H5A	120.4	C8—C13—H13A	120.0
C7—C6—C5	119.9 (6)	C9—C14—H14C	109.5
C7—C6—H6A	120.1	C9—C14—H14B	109.5
C5—C6—H6A	120.1	H14C—C14—H14B	109.5
C6—C7—C2	121.6 (6)	C9—C14—H14A	109.5
C6—C7—H7A	119.2	H14C—C14—H14A	109.5
C2—C7—H7A	119.2	H14B—C14—H14A	109.5
C9—C8—C13	121.6 (5)	C1—N1—C8	122.0 (4)
C9—C8—N1	118.8 (5)	C1—N1—H1	118 (4)
C13—C8—N1	119.6 (5)	C8—N1—H1	120 (4)
			
O1—C1—C2—C3	−59.1 (7)	C13—C8—C9—C10	−2.0 (7)
N1—C1—C2—C3	122.1 (5)	N1—C8—C9—C10	−179.2 (4)
O1—C1—C2—C7	120.7 (5)	C13—C8—C9—C14	177.8 (5)
N1—C1—C2—C7	−58.2 (6)	N1—C8—C9—C14	0.6 (7)
C7—C2—C3—C4	−3.2 (8)	C8—C9—C10—C11	−2.9 (9)
C1—C2—C3—C4	176.6 (4)	C14—C9—C10—C11	177.2 (6)
C7—C2—C3—Cl1	179.0 (4)	C9—C10—C11—C12	5.8 (10)
C1—C2—C3—Cl1	−1.2 (7)	C10—C11—C12—C13	−3.6 (10)
C2—C3—C4—C5	1.6 (8)	C11—C12—C13—C8	−1.3 (8)
Cl1—C3—C4—C5	179.5 (5)	C9—C8—C13—C12	4.2 (8)
C3—C4—C5—C6	0.2 (9)	N1—C8—C13—C12	−178.7 (4)
C4—C5—C6—C7	−0.3 (9)	O1—C1—N1—C8	3.0 (7)
C5—C6—C7—C2	−1.4 (9)	C2—C1—N1—C8	−178.3 (5)
C3—C2—C7—C6	3.1 (8)	C9—C8—N1—C1	−126.3 (5)
C1—C2—C7—C6	−176.7 (5)	C13—C8—N1—C1	56.4 (6)

### Hydrogen-bond geometry (Å, º)

**Table:**

*D*—H···*A*	*D*—H	H···*A*	*D*···*A*	*D*—H···*A*
N1—H1···O1^i^	0.86 (1)	2.00 (1)	2.853 (5)	171 (5)

Symmetry code: (i) *x*−1/2, −*y*+3/2, *z*.
